# A Hint on Vaccination

**Published:** 1817-08

**Authors:** F. Bush

**Affiliations:** Surgeon.


*For the London Medical and Physical Journal.*

*A Hint on Vaccination;* by Mr. F. Bush, Surgeon.

THE occurrence of small-pox twice in the same subject is a fact well established; but, I am not aware of its having attacked the same individual three times; that is, notwithstanding a person who has been once subjected to variola is commonly rendered insusceptible of the disease, yet the same subject may be twice infected, and go through the regular stages of variolous fever; but it has not, as far as I am acquainted with the subject, been noticed, that susceptibility to small-pox exists after the constitution has gone through it *twice.*

The occurrence of small-pox after vaccination is exactly analogous to secondary small-pox taking place after small-pox; the small-pox is unquestionably rendered milder by the previous vaccination, and, from its rare occurrence after vaccination, the susceptibility to the disease is generally done away. Reasoning from the analogy of the effect of the two diseases on the constitution, I am led to conclude, that, if the same individual was subjected to vaccine inoculation twice, or it might be done even three times, it would destroy the susceptibility to small-pox entirely.

*Frome; April* 26, 1817.

